# Comparative Analysis of Static and Dynamic Mechanical Behavior for Dry and Saturated Cement Mortar

**DOI:** 10.3390/ma12203299

**Published:** 2019-10-11

**Authors:** Ayyaz Mustafa, Mohamed A. Mahmoud, Abdulazeez Abdulraheem, Sarfaraz A. Furquan, Ayman Al-Nakhli, Mohammed BaTaweel

**Affiliations:** 1 Centre for Integrative Petroleum Research, College of Petroleum Engineering and Geosciences, King Fahd University of Petroleum and minerals (KFUPM), Dhahran 31261, Saudi Arabia; ayyaz.naim@kfupm.edu.sa; 2 Petroleum Engineering Department, College of Petroleum Engineering and Geosciences, King Fahd University of Petroleum and minerals (KFUPM), Dhahran 31261, Saudi Arabia; 3 Mechanical Engineering Department, King Fahd University of Petroleum and minerals (KFUPM), Dhahran 31261, Saudi Arabia; sfurquan@kfupm.edu.sa; 4 EXPEC ARC, Saudi ARAMCO, Dhahran 31311, Saudi Arabia; ayman.nakhli@aramco.com (A.A.-N.); MOHAMMED.BATAWEEL@aramco.com (M.B.)

**Keywords:** water saturation effects, mechanical properties, compressive strength, dynamic mechanical behavior, impact resistance, fracture resistance, failure pattern

## Abstract

Deformational and breakage behaviors of concrete and cement mortar greatly influence various engineering structures, such as dams, river bridges, ports, tunnels, and offshore rig platforms. The mechanical and petrophysical properties are very sensitive to water content and are controlled by the liquid part in pore spaces to a large extent. The objective of this paper is to investigate the water saturation effect on the strength characteristics and deformability of cement mortar under two loading conditions, static and dynamic compression. A set of cement mortar samples was prepared and tested to study the mechanical behavior in dry and saturated states. The first part of the research incorporates the study of static mechanical properties for dry and brine-saturated cement mortar through uniaxial compressive strength tests (UCS). Second, drop-weight impact experiments were carried out to study the dynamic mechanical properties (impact resistance, deformation pattern, and fracture geometry) for dry and saturated cases. The comparative analysis revealed that water saturation caused substantial changes in compressive strength and other mechanical characteristics. Under static loading, water saturation caused a reduction in strength of 36%, and cement mortar tended to behave in a more ductile manner as compared to dry samples. On the contrary, under dynamic loading conditions, water saturation resulted in higher impact resistance and fracture toughness as compared to dry conditions. In addition, fractures could propagate to smaller depths as compared to dry case. The study will help resolve many civil, mining, and petroleum engineering problems where cement structures undergo static as well as dynamic compression, especially in a hydraulic environment where these structures interact with the water frequently. To the best of our knowledge, the effect of water saturation on the dynamic mechanical properties of cement mortar has not been well understood and reported in the literature.

## 1. Introduction

Various concrete and masonry structures are constructed in a water environment and frequently come in contact with water, such as dams, ports, offshore rig platforms, the foundation of river bridges (piles and piers). The porous nature of concrete/cement and capillary pressures allows the water to penetrate inside it through pores and microfractures. Mechanical properties and strength characteristics are significantly affected due to the ingress of water into concrete [1,2,3,4]. Concrete structures constructed in the hydraulic environment also experience dynamic loadings, such as hydrodynamic pressures and earthquakes. Under dynamic loadings, concrete tends to deform in a different manner compared to static loads. Furthermore, the strength and mechanical properties of concrete are greatly influenced by the environment [5,6,7]. The durability and sustainability of concrete structures are greatly influenced by the strength characteristics and mechanical properties. Therefore, it is critically important to completely understand the mechanical behavior under different environments (dry and saturated) for the sustainability of concrete structures. The changes in mechanical properties of concrete/cement in the presence of water are mainly controlled by the mechanical and chemical effects of water [8,9].

Zhang et al. [10] studied the static mechanical properties for dry and saturated concrete samples and observed that tensile strength, compressive strength, and fracture toughness were reduced by 45.39%, 40.08%, 57.08%, respectively as a result of water saturation [10]. The water saturation effect on strength characteristics of concrete was examined experimentally, and reduction in tensile strength by 25% was observed as compared to dry concrete [11]. Dry concrete exhibits comparatively higher compressive and tensile strength as compared to saturated/immersed concrete. The degree of strength reduction increases with the duration of immersion [12]. For instance, compressive strength was determined for the concrete samples after immersion in water for different durations to achieve different degrees of saturation. The compressive strength was reduced by 17.45% for the sample with 75% water saturation and 33.79% for the sample with 100% water saturation [13]. Reduction in fracture toughness of concrete was observed based on experiments done at a low loading rate [14]. Tensile strength was determined for dry and immersed samples of concrete by Cadoni et al. [15]. The results exhibited a 7.62% reduction in the magnitude of tensile strength due to water saturation.

The experimental investigation on dry and saturated concrete samples revealed a significant decrease in compressive and tensile strength after immersion in water for 48 days. The strength characteristics reflected the pronounced decrease in tensile and compressive strength by 11.41% and 4.5%, respectively [16]. The experimental investigation exhibited a decrease in the flexural strength of concrete due to the increase in water content. Substantial changes in the mechanical properties of concrete are caused by water in two aspects: the mechanical effect of water and corrosive chemical action of water [17,18,19]. A series of experiments was performed to investigate the strength and strain rate dependency on water saturation. It was concluded that the presence of water causes a significant increase in strain rate and decreases the time to reach failure as compared to the results obtained in the dry state [20]. 

Several hypotheses explaining the effects of water saturation on properties of porous material, such as capillary tension decrease, fracture energy reduction, chemical and corrosive deterioration, frictional reduction, and decrease in effective stresses due to an increase in pore fluid pressure [21]. Several studies reported a decrease in uniaxial compressive strength (UCS) of 90% due to the increase in water content of 33% [22,23]. The significant influence of water content on fracture toughness and stiffness was observed due to chemical and physical deterioration. Fracture toughness was reduced by 43.1%, 33.3%, and 19.6% upon increasing the water saturation from zero to 3.5%, 2.0%, and 1.0%, respectively, which revealed a substantial change in deformability behavior [24]. Sandstones tend to fail in a more ductile manner with the release of a lesser amount of elastic energy as compared to the dry state due to the softening effect of water. Crack propagation exhibited a decreasing trend with an increase in water saturation. Crack propagation is faster in dry rock with rapid failure compared to the wet condition [24]. Porous materials tend to behave in a ductile manner at high moisture content and exhibit shear and brittle failure at low moisture content [25].

Under dynamic loading, the failure strain rate and fracture toughness of concrete are greatly influenced by the impact velocity and bedding planes angle [26]. An increase in dynamic fracture toughness has been observed as the impact velocity and loading rates increase. However, it starts decreasing after damage strain reaches its maximum due to high impact velocity [26]. Yin et al. [27] studied dynamic fracture characteristics at different loading rates on granite samples at different treatment temperatures using a split Hopkinson pressure bar (SHPB). An increasing linear trend has been observed for dynamic fracture toughness when loading rates increase at the same treatment temperature [27]. The dynamic fracture toughness shows a declining trend with increasing treatment temperature, and fracture resistance deteriorates. However, loading rates have more dominant effects on fracture characteristics as compared to temperature [27]. Dynamic mechanical properties are essential for strength characterisation of the material. It is essential to determine the dynamic strength characteristics of the rocks, but there is a lack of information in the literature on the dynamic mechanical behavior of rocks under both saturated and dry conditions. Several studies reported the influence of water saturation on the strength characteristics of different rocks types; however, most illustrated the strength and elastic parameters under static load [28,29].

A number of testing systems have been established by the International Society of Rock Mechanics since its establishment to study mechanical properties; however, most of them work under static loading conditions. Static mechanical testing functions at lower frequencies (less than tens Hertz generated by stress impulse of low amplitude) and strain rate is much lower as compared to practical engineering applications [30]. Dynamic loading usually works at stress pulses with high amplitude and a short time period. It is essential to perform a stability analysis of concrete structures under dynamic loading conditions, and mechanical properties are the key input for the stability assessment. Unfortunately, no standard has been set until now to determine the mechanical and strength characteristics under dynamic loadings [31]. The deformation behavior and fracture pattern under dynamic loads (high strain rate) are essential for many applications [32]. Fracture initiation and propagation under static compression loads have been reported in the literature; however, failure characteristics and deformation patterns are also strongly influenced by the dynamic compression load, especially in the presence of micro-fractures or defects [33,34].

Generally, several impact tests are implemented to illustrate the impact resistance and relative brittleness of concrete and other construction material [35,36]. However, no standard test method has been declared to study the dynamic properties of materials, as no statistical data are available on the comparison of results. A drop-weight impact test was proposed by the American concrete institute (ACI) committee (1996) [37] to study the impact resistance and fracture toughness of fiber concrete and cement. The test is extensively used because of its simplicity and cost effectiveness. However, the results of the drop-weight test are prominently scattered. 

To the best of our knowledge, all the reported cases for strength reduction are under static loading conditions. Many researchers investigated and reported the mechanical properties and strength characteristics of cement and concrete under static loading. However, the study of mechanical properties and failure characteristics under high loading rates is not well understood and explained. Limited literature is available on the effect of water saturation on impact resistance and brittleness under dynamic loading. This study incorporated the effect of water saturation on static strength as well as dynamic mechanical properties (impact resistance, impact energy, deformation pattern, and fracture geometry) under impact loading system (Instron weight falling system).

The objective of this paper is to determine and understand the sensitivity of cement mortar towards water content under both dynamic and static compression. Cement mortar responds differently under both dynamic and static compression loadings. Strength is very sensitive to water content and decreases considerably from the dry to saturated state. On the other hand, impact resistance and fracture toughness increase for saturated samples under dynamic loading. 

## 2. Materials and Methods 

The research aims to study the static and dynamic mechanical properties under dry and saturated states. The dynamic and static loading approaches were implemented to examine the role of water saturation on mechanical properties and strength characteristics. A uniaxial compressive strength (UCS) test was performed as per American Society for Testing and Materials (ASTM) standards D7012 using the rock mechanics system ‘AutoLab 1500’ for four dry and four saturated samples. Compressive strength was measured without confining pressure, and Young’s modulus was calculated at 50% of maximum load. Instron dynatup impact testing system (Instron 9250G, Norwood, MA, USA) was used to determine the impact resistance and fracture toughness of cement mortar under dynamic conditions. Fracture pattern, peak deflection, maximum load, and maximum energy were analysed for dry and saturated samples. 

### 2.1. Sample Preparation 

**Figure 1:**
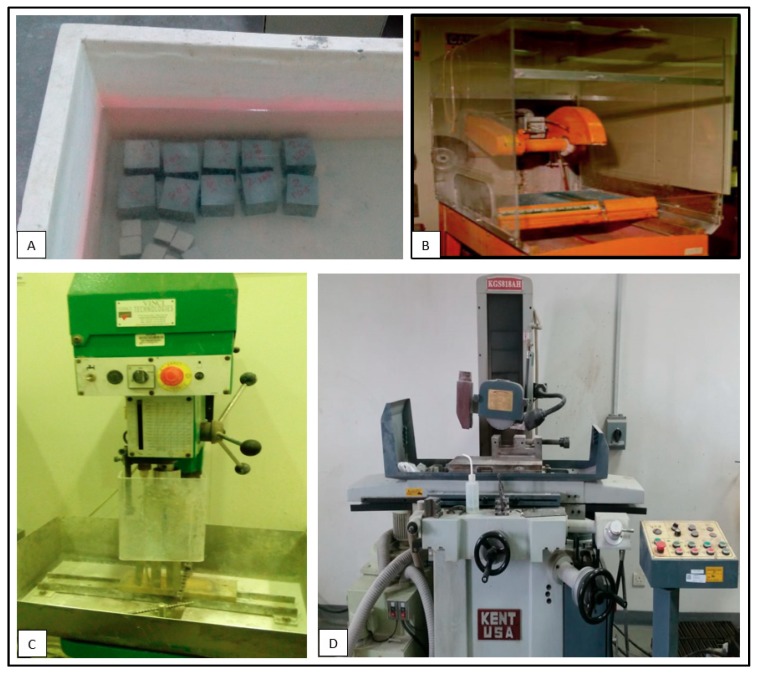
Sample preparation phases of cement mortar (**A**) Curing, (**B**) Cutting, (**C**) Coring, (**D**) End face grinding.

**Figure 2:**
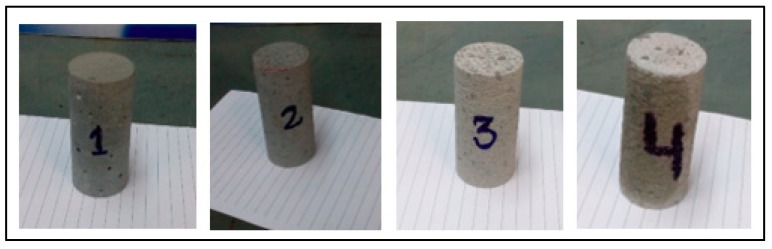
Cement mortar samples (length: 2.0˝and dia: 1.0˝) prepared for experimental work.

Cement mortar samples were cast and cured for seven days with the mix design of a 1:1 cement sand ratio for the experimental work. The porosity and permeability of cement mortar were measured as 12.5% and 1.1 milli Darcy, respectively. Core plugs were prepared by coring and end-face grinding processes (Figure 1). Cement mortar core plugs are shown in Figure 2. Half of the samples were fully saturated with 3% KCl solution (brine) at a pressure of 500 psi using a saturator. The rest of the samples were air-dried. The samples have a length to diameter ratio of 2:1 in accordance with ASTM C39 standards. The samples were then polished from both ends with a tolerance of 0.0025” to maintain parallelism. 

### 2.2. Uniaxial Compression Test (ASTM C39)

Uniaxial compressive strength tests were carried out as per the ASTM C39 standard. Tests were conducted on NER AutoLab 1500 triaxial frame (New England research, White River Junction, VT, USA) using linear variable differential transformers (LVDT, White River Junction, VT, USA) to measure sample deformation in both axial and radial directions. Metals deform equally regardless of the strain rate, while rocks are strain-rate dependent. Therefore, stress was applied at a standard constant strain rate of 2 mm/h. The elastic properties were calculated at 50% load, which is the best practice to obtain accurate results.

### 2.3. Impact Test

A dart impact tester (Instron 9250G) (Instron, Norwood, MA, USA), instrumented with different sensors, was used to determine the fracture toughness and impact resistance of the dry and saturated cement mortar. 

**Figure 3:**
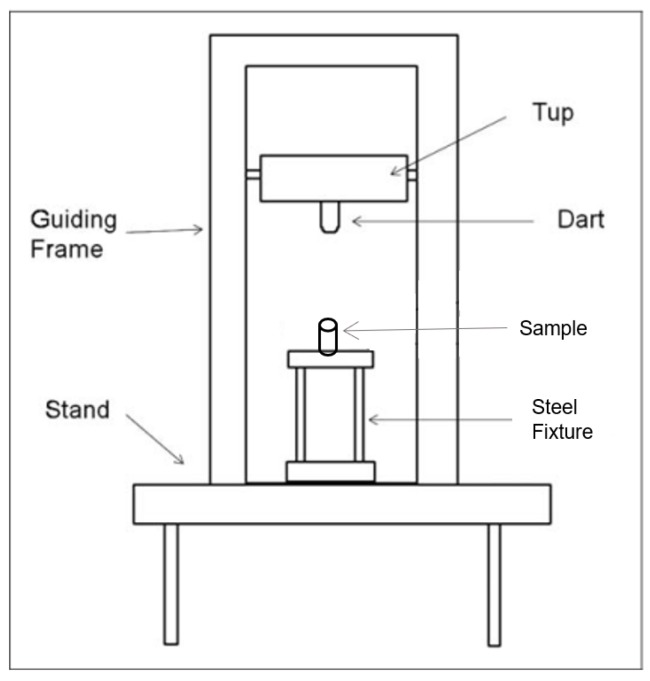
Schematic of the impact test machine.

A computer system having data acquisition and analysis software was connected to the Instron Dynatup 9520G (Instron, Norwood, MA, USA). The equipment consists of a free-falling drop-weight system with a pneumatic system for brakes. Figure 3 shows the schematic diagram of the general drop-weight impact test equipment. A steel fixture having grooves for holding 1-, 1.5-, and 2-inch diameter core samples was designed and manufactured. 

The system software measured the data of load vs. time and instantaneous velocity at the time of impact on the specimen. These measured parameters were further used to calculate the specimen deflection, energy absorbed by the specimen, and tup velocity. Data acquisition hardware used the instrumented tup and record the load vs. time data for each impact test. Photoelectric-diode and flag system were used to determine the impact velocity just before the impact of load on the specimen. Another option is the method editor for setting the velocity of impact, which is used by software for calculating other parameters.

#### 2.3.1. Impact Velocity

Typically, a velocity measuring system uses a flag on the drop weight/pendulum to commence data acquisition and to record impact velocity. The velocity of the drop weight/pendulum as the flag exits the detector is found. The following equations describe the flag velocity calculation [38]:

Velocity: v_flag_ = (w_flag_/t_flag_) + ½ g (t_flag_) (1)

where g = acceleration of gravity (9.81 m/s^2^) or (32.2 ft/s^2^)

W_flag_= effective flag width = distance from 1st leading edge to 2nd leading edge (1 cm)
t_flag_ = Time from the moment the 1st leading edge obstructs the beam to the moment the second leading edge obstructs the beam (measured in seconds).
v_flag_ = Velocity of the flag as it exists in the photo-diode detector

To find the impact velocity, we substitute t_imp_ for t_flag_ in Equation (1), and we obtain:

V_imp_ = (w_flag_/t_imp_) + ½ g (t_imp_) (2)

t_imp_ = elapsed time between the 2nd leading edge obstructing the beam to the moment just before impact.

#### 2.3.2. Deflection, Velocity, and Energy

The remaining parameters of deflection, velocity, and energy are calculated using standard equations of motion for an object traveling in a straight line being subjected to an opposing force (Equations (3)–(6)). Using this method, expressions are derived for velocity, deflection, and absorbed energy. The total force acting on the drop weight/pendulum is assumed to be the sum of the resistive force offered by the specimen w(t) and the force of gravity. Therefore, data assumption starts at t = 0:

(3) F(t)=mg−w(t)

(4) $$ac\left(t\right)=\frac{F\left(t\right)}{m}=g-\frac{w\left(t\right)}{m}$$

(5) $$V\left(t\right)=ac\left(t\right)dt=gt-\frac{1}{m}\times w\left(t\right)dt$$

(6) $$D\left(t\right)=V\left(t\right)dt=\frac{1}{2}gt^{2}-\frac{1}{m}\times w\left(t\right)dt$$

*w(t)* = The load applied to the drop weight/pendulum by the specimen at an arbitrary time ‘t’.
*m *= The total mass of the drop weight/pendulum.
*g* = Gravitational acceleration (9.81 m/s^2^) or (32.2 ft/s^2^).
*F(t)* = Resultant force acting on the drop weight/pendulum.
*ac(t)* = Resultant acceleration of drop weight/pendulum at any time ‘t’.
*V(t)* = Velocity of drop weight/pendulum at any time ‘t’.
*D(t)* = Deflection (Position) of the drop weight/pendulum at any time ‘t’.
*K(t)* = Kinetic energy of drop weight/pendulum at any time ‘t’.
*P(t)* = Potential Energy of the drop weight/pendulum at any time ‘t’.
*Ea(t)* = Amount of energy absorbed by the specimen up to point in time ‘t’.
*E(t)* = Total amount of energy of the drop weight/pendulum and specimen system at any time ‘t’.

From the conservation of energy principle applied to the drop weight/pendulum and specimen system, the total energy is computed as follows:

Energy (total): E(t) = P(t) + K(t) + Ea(t) = constant(7)

At any time ‘t’:

(8) $$Ea\left(t\right)=\left(\frac{m}{2}\right)\times (v^{2}imp-v^{2}\left(t\right)+mgD\left(t\right)$$

## 3. Results and Discussions

The comparative analysis was done for compressive strength parameters for both saturated and dry cases under static and dynamic loading. The UCS experimental results exhibited remarkably different compressive strength characteristics and elastic properties for both the dry and saturated cases. Likewise, dynamic compression test results revealed the effect of water saturation in terms of considerable different impact resistance and fracture toughness in both cases. In the case of static loading, a substantial reduction in unconfined compressive strength and Young’s Modulus was observed for water-saturated mortar (Table 1). They exhibited ductile behaviour due to an increase in pore pressure. Pore pressure is one of the critical factors that causes changes in the mechanical behaviour of porous materials, such as cement, concrete, and rocks. Pore-water interacted with solid grains of cement mortar and caused its strength and elastic behaviour to change. On the contrary, results are quite the opposite under dynamic loading, where high loading rates caused the impact resistance and fracture toughness to increase in the presence of water content. More resistance was observed in terms of maximum energy and load for creating fractures in saturated samples as compared to dry cement mortar samples, as shown in Table 2. 

### 3.1. Static Compression Test Results

**Figure 4:**
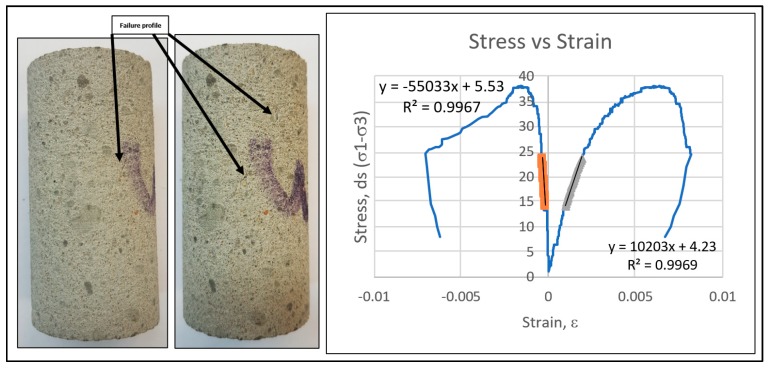
Uniaxial compressive strength test results for dry samples.

**Figure 5:**
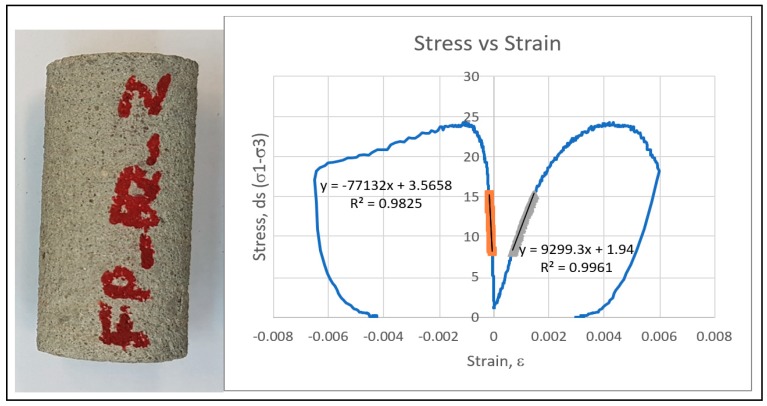
Uniaxial compressive strength test results for saturated samples.

**Figure 6:**
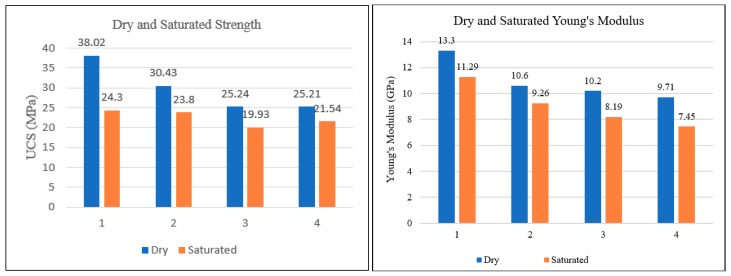
Comparison of uniaxial compressive strength and Young’s modulus for both dry and saturated cases.

The compressive strength of saturated cement mortar was reduced by 36% compared to dry mortar. Higher stiffness and resistance to failure was observed in dry cement mortar in terms of higher Young’s modulus as compared to saturated case. The UCS results and failure profile for dry cement mortar samples are shown in Figure 4. The results exhibited higher strength (38.02 MPa) and stiffness with Young’s modulus of 13.3 GPa. The UCS and Young’s modulus of the same sample reduced to 24.30 MPa and 11.29 GPa, respectively, exhibiting the weakening effects of water. The UCS results for the saturated cases are shown in Figure 5. The saturated samples exhibited ductile behaviour with no prominent failure occurring under static loading, as reflected by the stress–strain relationship. A comparison of strength characteristics and elastic parameters for saturated cases are shown below in Figure 6.

The resistance against elastic deformation and failure was noticeably lower in saturated samples as compared to dry case. Dry cement mortar is able to withstand higher stress without being deformed and fractured as reflected by stress-strain relationship comparison (Figure 4 and Figure 5). The water weakening effect deteriorates the compressive strength and stiffness of cement mortar (Figure 6). The dry cement mortar tended to fail in a less ductile/brittle manner with prominent failure patterns developed under uniaxial stress, as shown in Figure 4. On the contrary, the saturated cases revealed more ductile failure/deformation. Therefore, no apparent failure pattern or fracture was observed in the sample even after deformation, as shown in the stress–strain relationship profile (Figure 5).

One of the evident reasons for the deformation of saturated samples without apparent fracture is the gradual collapse of grain framework or cracks generation inside the samples. Furthermore, the saturated mortar samples reflected low lateral strain exhibiting weakening of the grain’s framework due to the presence of brine in pores. In the dry case, mortar responded to strain by fracturing. In contrast, ductile failure in saturated mortar explains the strain accommodation by plastic deformation. Thus, in ductile failure, more energy was absorbed due to more plastic strain. Failure properties (internal friction and cohesion) of cement mortar were strongly influenced by the water-cement interaction that may lead to the different failure phenomena in both dry and saturated mortar. Ductile failure in saturated samples was predominantly caused by the reduction in the angle of friction. High values of coefficient of determination (R^2^) of linear regression (0.98 and 0.99) indicated high accuracy and reliability of the obtained data points of UCS.

### 3.2. Impact Load Results

**Figure 7:**
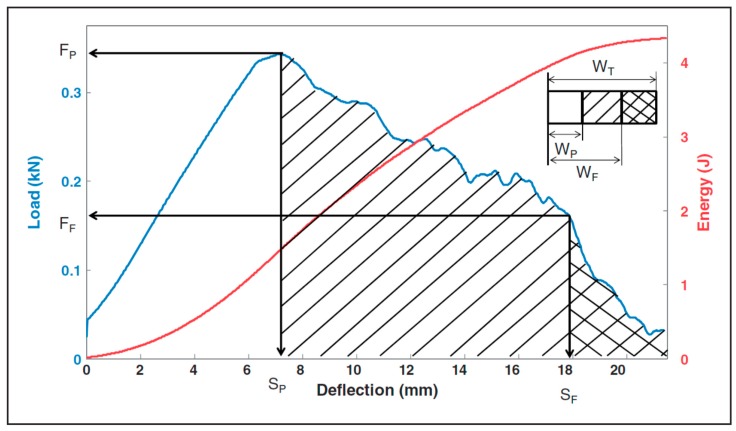
Deformation force diagram for an impact load tested specimen [39].

Impact loading through a dynamic compressional test was performed on both unsaturated and saturated cement mortar samples. Cylindrical shaped cement mortar samples having dimensions of 1-inch diameter and 2-inch length were subjected to dart impact tests. The effect of two different environmental conditions on the dart impact behavior of cement mortar samples was studied. The samples of the first condition were tested in the dry state. The samples of the second condition were kept in brine solution and upon saturation were impact tested. The dart was made to drop from a height on the cement mortar sample placed concentric to the falling dart. The velocity of the striking dart on the flat end of the sample was 2 m/s. Two test samples were tested under dry conditions and three test samples from saturated conditions. The impact resistance behavior of these samples was analysed. The deformation versus force plot, as shown in Figure 7, illustrates the important terminologies, such as peak force, peak deformation, energy at peak force, failure force, failure deformation, energy at failure force, and total energy used in the impact load test, and are described elsewhere [39].

**Figure 8:**
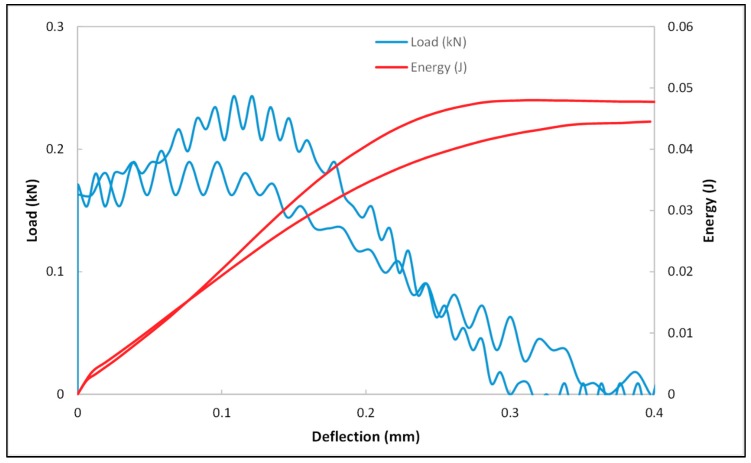
Load and deflection plot of dry cement mortar samples.

**Figure 9:**
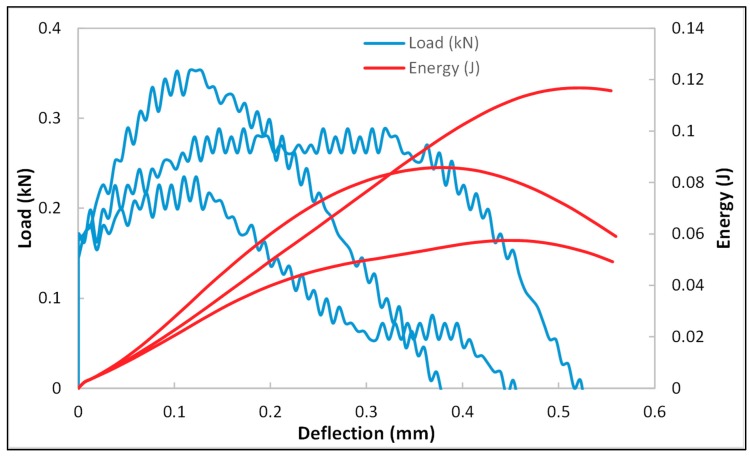
Load and deflection plot of brine-saturated cement mortar samples.

The load/energy vs. deflection plots (Figure 8 and Figure 9) of dry and brine-saturated cement mortar samples indicated that the samples were brittle. The average impact test results are presented in Table 2 and Table 3. Both samples exhibited brittle behavior due to the appearance of a small deflection after the maximum force peak. The dry samples were tested at two different velocities of 2 m/s and 3 m/s, whereas saturated samples were tested at 2 m/s. For comparison between dry and saturated samples, only the results from a velocity of 2 m/s will be considered for dry and saturated samples. Both the dry and brine-saturated cement mortar samples exhibited a sharp spike in the load at 2 m/s dart velocity. Similarly, a spike in load was observed at 3 m/s in dry samples, indicating that the samples are rigid. The peak force required for fracturing the samples was higher in saturated samples (0.292 kN) as compared to the dry samples (0.2433 kN). The amount of deflection at peak force for saturated samples was 0.1198 mm, and for dry samples was 0.1084 mm. The region between peak deformation and failure deformation describes the fracture propagation behavior of the samples. The deflection at failure force for saturated samples was 0.4108 mm, and for dry samples was 0.2613 mm. For the region between peak and failure deformation, it was observed that the fracture propagated at a faster rate in dry samples as compared to saturated samples. Further, the total energy for fracturing saturated samples (0.0867 J) was higher compared to dry samples (0.0479 J).

**Figure 10:**
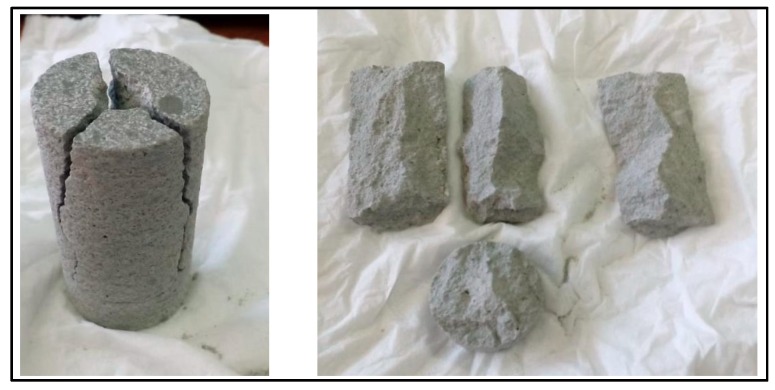
Fractured mortar sample after dynamic compression impact loading test (Dry Case).

**Figure 11:**
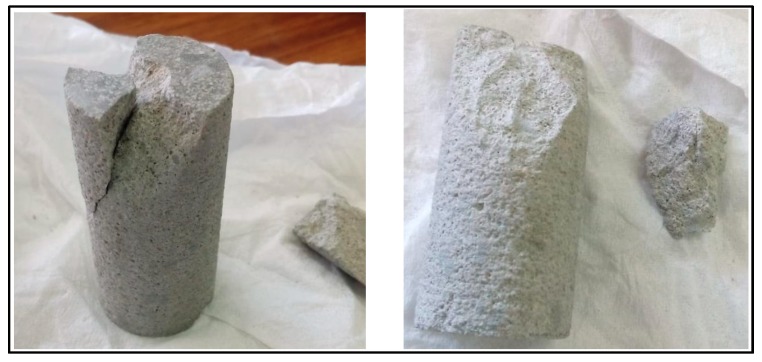
Fractured mortar sample after dynamic compression impact loading test (Brine-saturated case).

The fragments of the fractured samples were carefully analysed, and it was observed that in the case of dry samples, the fracture propagates deeply into the material along the longitudinal axis in the direction of the falling dart (Figure 10). Further, it gets shattered into three pieces of almost equal size. On the other hand, the fracture in the saturated samples propagates to smaller depths along the direction of falling dart before moving outward in the transverse direction (Figure 11). Moreover, the fracture breaks only a segment on the top of the sample, and the rest of the material was intact from the top to the bottom of the sample. 

The total impact energy for saturated samples was higher compared to the dry sample. The saturated samples offered higher resistance to fracture propagation along the direction of dart impact compared to the dry sample. The energy of impact load bounced back because pore pressure was generated in the presence of water. A higher amount of energy was required to reach the failure/deformation in the saturated case because both solid and fluid parts play an active role in responding to the applied impact load.

## 4. Conclusions

In different engineering applications, masonry and cement structures are exposed to static as well as dynamic compression loadings. Moreover, strength characteristics and elastic properties are strongly influenced by the environment, especially the hydraulic environment. The dynamic mechanical behavior in a saturated state is not well understood and explained. Major conclusions drawn from the experimental investigation of mechanical properties and strength characteristics under static and dynamic loads are mentioned as follows:

- Sustainability of cement mortar structures was strongly influenced by the water exposure in terms of a substantial change in compressive strength characteristics and elastic mechanical parameters (Young’s Modulus and Poisson’s ratio) due to ingress of water into the pores. The water weakening effect deteriorated the compressive strength and stiffness of cement mortar.
- Under static loading, water saturation caused a reduction in the strength of cement mortar of 36% and tended to behave in a more ductile manner as compared to dry samples. On the other side, dry cement mortar exhibited higher strength and deformed in a less ductile or brittle mode. Higher resistance to failure/deformation was noticed for dry cement mortar in terms of higher Young’s modulus in comparison with the saturated case. Saturated cement mortar exhibited a ductile mode of deformation with no evident failure pattern appearing under static load due to saturation effects.
- Under dynamic loading, saturated cement mortar are proved to be stronger in terms of higher impact resistance and fracture toughness as compared to dry mortar. In comparison with dry cement mortar, higher resistance to fracture propagation along the direction of dart impact was offered by saturated samples. A relatively higher amount of impact energy was required to reach the failure/deformation in the saturated case because both solid and fluid parts play an active role in responding to the applied load. The energy of impact load bounced back due to generated pore water pressure. Furthermore, the fracture could propagate to smaller depths along the direction of falling dart before moving outward in the transverse direction as compared to dry mortar.
- Mechanical behaviour for dry and saturated cement mortar has not been completely understood and explained in the literature, particularly under dynamic loads. Hence, this research will effectively contribute to providing a better understanding of the mechanical behavior under static and dynamic loads for two different aspects: dry and saturated states.

## Figures and Tables

**Table 1:** Uniaxial compressive strength and Young’s modulus comparison of dry and saturated samples.

Sample	Strength (Dry Sample) (MPa)	Strength (Saturated) (MPa)	Percentage Decrease (%)	Young’s Modulus (Dry) (GPa)	Young’s Modulus (Saturated) (GPa)	Percentage Decrease (%)
1	38.02	24.30	36.1	13.3	11.29	15.1
2	30.43	23.80	21.7	10.6	9.26	12.6
3	25.24	19.93	21.0	10.2	8.19	19.7
4	25.21	21.54	14.6	9.71	7.45	23.3

**Table 2:** Impact test results of dry specimens.

Test No	Maximum Load (kN)	Time to Max Load (ms)	Impact Velocity (m/s)	Total Energy (J)	Total Time (ms)
2	0.1987	0.0153	3.1212	0.0442	0.1129
5	0.2433	0.0488	2.0096	0.0479	0.1404
Average	0.221	0.032	2.5654	0.0461	0.1266

**Table 3:** Impact test results of brine-saturated specimens.

Test No	Maximum Load (kN)	Time to Max Load (ms)	Impact Velocity (m/s)	Total Energy (J)	Total Time (ms)
1	0.2884	0.0671	2.0057	0.1168	0.2441
2	0.3525	0.0458	2.0298	0.0858	0.177
3	0.235	0.0488	2.0397	0.0575	0.2075
Average	0.292	0.0539	2.0251	0.0867	0.2096
Standard Deviation	0.0588	0.0116	0.0175	0.0297	0.0336
